# Application of the Demirjian Classification Scheme in a Selected South African Population: A Retrospective Analysis

**DOI:** 10.1155/ijod/9931755

**Published:** 2025-10-31

**Authors:** S. Ishwarkumar-Govender, K. S. Satyapal, P. Pillay

**Affiliations:** ^1^Department of Human Anatomy and Physiology, Faculty of Health Sciences, University of Johannesburg, Johannesburg, South Africa; ^2^Department of Clinical Anatomy, School of Medicine, University of KwaZulu-Natal, Durban, South Africa

**Keywords:** Demirjian method, dental age, forensic age estimation, panoramic radiography, tooth development

## Abstract

**Introduction:**

In recent years, forensic age estimation gained significance owing to the rising incidence of violence, motor vehicle accidents, and mass disasters. Human dentition is a key component in biological profiling, with factors such as genetics, sex, and population affinity influencing its development and eruption. Dentition development is currently recognized as one of the most effective methods for estimating chronological age in young individuals. The Demirjian method is widely used in pediatric dentistry. However, its accuracy varies across different populations and/or regions. This paper explores the applicability of the Demirjian method in estimating dental age in a select KwaZulu-Natal sample of South Africa.

**Materials and Methods:**

This retrospective cross-sectional study utilized 480 digital panoramic radiographs aged between 5.00 and 16.99 years (*n* = 480:240 South African Black and 240 South African Indian). Consecutive sampling was implemented, and radiographs were evenly distributed among the aforementioned population groups, including sexes and all age cohorts. Using the CareStream (CS) Imaging Software, each digital panoramic radiograph was assessed using the Demirjian method.

**Results:**

This study found that the Demirjian method overestimates dental age for the selected South African sample. It is recommended that population-specific norms should be utilized when estimating dental age in the South African population groups to avoid over or underestimation of dental age. In addition, a combination of more than one dental age estimation method should be utilized to obtain more accurate results.

**Conclusion:**

To conclude, this study's findings enhance the existing literature and may prove valuable in forensic and medicolegal contexts.

## 1. Introduction

In recent years, the importance of forensic age and sex estimation has surged due to the rise in incidents involving violence, motor vehicle accidents, and mass disasters [1–4]. “South Africa has the fifth-highest crime rate in the world,” with a notably high rate of violent crimes (assaults, rape, and homicides) (https://worldpopulationreview.com/country-rankings/crime-rate-by-country). KwaZulu-Natal is not only ranked as having the third-highest crime rate in South Africa [5], but it is also prone to natural disasters (i.e., landslides, flooding, and tornadoes). In April 2022, catastrophic floods led to the loss of more than 400 lives [6], and in June 2024, a tornado swept through the province, resulting in 12 fatalities [7].

Forensic dentistry focuses on dentition analysis, which is essential for identifying individuals and estimating age, whether they are living or deceased [4, 8–10]. Utilizing radiography for dental age and sex estimation is a critical aspect of forensic dentistry [10–13]. Despite the availability of various dental age assessment methodologies, the radiological approach stands out for its simplicity, noninvasiveness, and cost-effectiveness [14–16].

Teeth, being exceptionally durable, chemically stable, and highly mineralized, are invaluable in forensic studies, particularly for age estimation [17]. The development and eruption of teeth serve as valuable chronological markers for life-history events, aiding in the assessment of maturity and assignment of chronological age [17, 18]. Estimating dental age through mineralization phases is a commonly used method to determine an individual's chronological age [13, 19], leveraging the developmental stages of dentition [9]. This involves scoring different teeth and averaging the results to derive an estimated dental age, although its applicability is limited to permanent dentition [9].

The Demirjian et al. [20] method is based on a French–Canadian sample's seven permanent dentitions in the left mandibular quadrant. This method describes eight stages of tooth development. Stage A: Beginning of calcification to Stage H: Apical of the root is closed [20]. Each developmental stage is associated with a self-weighted score for females and males, and the sum of the self-weighted scores for the seven mandibular dentitions (excluding third molars) correlates with an estimated dental age in the conversion of maturity score tables for females and males [20]. However, there have been debates regarding the applicability of the Demirjian method in different population groups, with some studies documenting an underestimation of dental age [21–23], while others have recorded an overestimation of dental age [24–30]. In addition, Yang et al. [31] reported an underestimation of dental age in females and an overestimation in males, with no statistically significant difference recorded between the chronological and dental ages in both groups.

In South Africa, there are nine provinces with distinct, diverse population groups, viz. Black, White, Indian, and Colored. The Demirjian method only assessed the Black South African population in one province, that is, Gauteng [32, 33], with a finding of overestimation in dental age. Since population-specific standards are variable, consideration should be given as the best estimates for age assessment [9]. Therefore, this study aimed to determine the applicability of the Demirjian Classification Scheme in a selected South African population within KwaZulu-Natal.

## 2. Materials and Methods

### 2.1. Sample and Study Design

This retrospective cross-sectional study analyzed 480 digital panoramic radiographs of individuals aged between 5.00 and 16.99 years (*n* = 480), comprising equal representation of South African Black (*n* = 240) and South African Indian (*n* = 240) participants. A consecutive sampling strategy was employed to ensure balanced distribution across population groups, sexes, and 1-year age cohorts (e.g., 10 radiographs per subgroup such as Black females aged 5.00–5.99 years). Population classification was based on “modern systems of racial classification,” which consider skin color and ancestry [34, 35]. The sample distribution reflects the demographic alignment of KwaZulu-Natal, where South African Black and South African Indian communities are predominantly located [36]. All demographic data were securely stored in password-protected documents to ensure confidentiality. The age of the individual was calculated by subtracting the date of birth from the date the panoramic radiograph was taken.

### 2.2. Selection Criteria

This study excluded all digital panoramic radiographs with missing (congenital or extracted) and/or impacted dentition as well as all panoramic radiographs with recognizable anomalies/pathologies or trauma in the maxillofacial region. The exclusion criteria also excluded distorted and/or blurred panoramic radiographs from this study.

### 2.3. Demirjian et al. [20] Method

Digital panoramic radiographs were evaluated using CS Imaging Software (Version 7.0.20), with dental age estimation conducted in accordance with the method outlined by Demirjian et al. [20], which examines tooth development stages in the left mandibular and maxillary quadrants. Each tooth was assigned a maturity score based on its developmental stage, and the scores were then summed [20]. These totals were referenced against Demirjian et al.'s [20] “Conversion to Maturity Score to Dental Age 7 Teeth” tables, stratified by sex, to estimate dental age. Approval for this study was obtained from the relevant institutional ethics committee (Reference number: BE 405/17). As a retrospective analysis of existing radiographs, gatekeeper permission was secured from the Manager of the participating Private Practice. To minimize bias, the first author remained blinded to all demographic data during the data collection process.

### 2.4. Observer Agreement

To ensure this study's intra and interrater reliability and validity, the first author and a second examiner reassessed and assessed 24 digital panoramic radiographs (5%) using the intraclass correlation coefficient test.

### 2.5. Statistical Analysis

The sample size was determined through power analysis using the “Raosoft” sample size calculator. At a 95% confidence level, a minimum of 385 participants was determined to be necessary to achieve adequate statistical power. Statistical analyses were conducted using R Studio for Windows (Version 4.0.2). The paired sample *t*-test was used to evaluate if there was a significant difference between chronological age and estimated dental age, with significance determined at *p*  < 0.05. The age prediction performance was evaluated using mean error (ME), calculated as the mean difference between chronological and estimated dental ages, to assess systematic bias. In addition, mean absolute error (MAE) was computed to quantify the magnitude of prediction errors and overall estimation accuracy.

## 3. Results

In this study, an overestimation of dental age was recorded for the selected sample using the Demirjian et al. [20] method (Tables 1–3). An overestimation of 0.66, 1.02, and 0.82 years was recorded in the maxilla, mandible, and both maxilla and mandible combined, respectively (Table 1). In this study, an overestimation of 0.56 and 0.83 years was recorded for the total female sample in the maxilla and mandible, respectively. While the total male sample had an overestimation of 0.76 years in the maxilla and 1.21 years in the mandible (Table 1). The South African Black female sample displayed an overestimation of 0.72 years in the maxilla and 0.99 years in the mandible, while their male counterparts had an overestimation of 0.93 years and 1.24 years in the maxilla and mandible, respectively (Table 1). In contrast, an overestimation of 0.38 years and 0.58 years in the maxilla and 0.66 years and 1.18 years in the mandible was recorded for the South African Indian female and male samples, respectively (Table 1). In this study, a statistically significant difference exists between the estimated dental age and chronological age (Table 1). Overall, the overestimation was greater in males than females for the individual age ranges in both population groups (Tables 2 and 3). Similarly, the overestimation was greater in the mandible than the maxilla for the individual age ranges in both sexes and population groups (Tables 2 and 3). Additionally, the study reported intraobserver and inter-observer reliability coefficients of 0.88 and 0.85, respectively.

## 4. Discussion

This study recorded that the Demirjian et al. [20] method overestimated dental age for the selected South African sample by −0.66 and −1.02 years using the dentition in the left quadrant of the maxilla and mandible regions, respectively (Table 1). However, when the dentition in the left quadrant of the maxilla and mandible were used together, an overestimation of 0.82 years was recorded in this study (Table 1). The overestimation in dental age reported in this study concurred with the findings of Koshy and Tandon [24]; Willems et al. [25]; Mani et al. [26]; Urzel and Bruzek [27]; Ambarkova et al. [28]; Moness Ali et al. [29]; Moca et al. [30]; Uys [32] and Esan and Schepartz [33]. However, some studies reported an underestimation of dental age [21–23]. Additionally, a statistically significant difference was found between the estimated dental age and chronological age in this study (Table 1).

Moreover, this study noted that the overestimation in dental age was lower in the South African female sample compared to their male counterparts for both population groups (Table 1). This finding correlates with the study by Moness Ali et al. [29] and Maco et al. [30], who also reported a lower overestimation of dental age in females compared to males. However, Esan and Schepartz [33] reported that South African Black males had a lower overestimation of dental age than South African Black females in the Gauteng region, viz. 0.85 years and 1.00 year, respectively. Han et al. [37] reported that regional differences may be attributed to environmental factors, viz., nutrition, dietary habits, and lifestyle, which influence tooth development as well as differences in sampling (sample size and number of individuals in different age groups–uneven vs. even number of individuals per age range) [37]. Furthermore, it is essential to recognize that secular changes may influence dental maturity, indicating that the timing of investigation can affect observed developmental patterns. For example, Poulsen and Sonnesen [38] found that Scandinavian children born between 2005 and 2010 exhibited significantly earlier dental maturation compared to those born between 1969 and 1973.

In addition, the overestimation in dental age was lower in the South African Indian male and female samples compared to the South African Black male and female samples (Table 1). Despite the Demirjian et al. [20] method being based on the dentition in the left mandibular quadrant, the lowest over-estimation in dental age for both sexes and population groups was recorded for the dentition in the left maxillary quadrant, followed by the combination of dentition from the maxillary and mandibular quadrants (Table 1).

Furthermore, the over-estimation in dental age was generally greater in males than females in the individual age ranges for population groups (Tables 2 and 3). This correlated with Hostiuc et al. [39] who report that “the smallest errors being found in the 12–14 and 15–15 years-old intervals” in their meta-analysis study. Moreover, Hostiuc et al. [39] elaborated that this method becomes unreliable for advanced age groups since “the values given for the dental age are outside the prediction interval”. In their meta-analysis, an underestimation in dental age was recorded for the 14–15-year-old age group. However, in the present study an overestimation in dental age was documented for these age ranges, while an underestimation in dental age was recorded for the 16.00–16.99 age range. This may be due to the population-specific differences, as Hostiuc et al. [39] stated that “if the study population is different from the population for which the method is used, there could be significant differences limiting the accuracy of the method.” Therefore, population-specific norms should be created and utilized for dental age estimation.

## 5. Limitation

This study was limited by the exclusion of the South African Colored and South African White population groups, as insufficient data prevented the conduct of reliable statistical analysis. However, this reflects the demographic makeup of KwaZulu-Natal, where South African Black and South African Indian populations are the majority [36].

## 6. Conclusion

The Demirjian et al. [20] method overestimated dental age in the selected South African sample from the KwaZulu-Natal region, with a statistically significant difference between the estimated dental age and chronological age. Therefore, this study recommends that population-specific (genetic) and geographical differences should be considered when employing dental age estimation methods, and population-specific norms should be utilized when estimating dental age in the South African population groups to avoid over or underestimation of dental age, as these international standards may not be designed for utilization in all population groups. In addition, a combination of more than one dental age estimation method should be utilized to obtain more accurate results. Subsequently, the findings of this study may assist in forensic and/or medicolegal cases.

## Figures and Tables

**Table 1:** Comparison of the chronological and estimated dental age using the Demirjian et al. [20] method (years).

Sample	Sample size (*n*)	Mean CA ± SD (years)	Mean DA for maxilla ± SD (years)	Mean error for maxilla (years)	Mean absolute error for maxilla (years)	*p*-Value for maxilla (years)	Mean DA for mandible ± SD (years)	Mean error for mandible (years)	Mean absolute error for mandible (years)	*p*-Value for mandible (years)	Mean DA for maxilla and mandible ± SD (years)	Mean error for maxilla and mandible (years)	Mean absolute error for maxilla and mandible (years)	*p*-Value for maxilla and mandible (years)
Black female	120	10.99 ± 3.48	11.71 ± 3.28	−0.72	0.89	<0.001	11.98 ± 3.09	−0.99	1.13	<0.001	11.82 ± 3.18	−0.83	0.95	<0.001
Black male	120	10.99 ± 3.53	11.92 ± 3.45	−0.93	1.07	<0.001	12.23 ± 3.40	−1.24	1.36	<0.001	12.07 ± 3.44	−1.08	1.20	<0.001
Indian female	120	11.04 ± 3.48	11.42 ± 3.33	−0.38	0.70	<0.001	11.70 ± 3.19	−0.66	0.91	<0.001	11.54 ± 3.25	−0.50	0.75	<0.001
Indian male	120	10.99 ± 3.48	11.57 ± 3.51	−0.58	0.80	<0.001	12.17 ± 3.35	−1.18	1.27	<0.001	11.86 ± 3.48	−0.87	0.99	<0.001
Total female	240	11.01 ± 3.47	11.57 ± 3.30	−0.56	0.79	<0.001	11.84 ± 3.14	−0.83	1.02	<0.001	11.68 ± 3.21	−0.67	0.85	<0.001
Total male	240	10.99 ± 3.50	11.75 ± 3.48	−0.76	0.94	<0.001	12.20 ± 3.37	−1.21	1.31	<0.001	11.96 ± 3.45	−0.97	1.10	<0.001
Total sample	480	11.00 ± 3.48	11.66 ± 3.39	−0.66	0.86	<0.001	12.02 ± 3.26	−1.02	1.17	<0.001	11.82 ± 3.33	−0.82	0.97	<0.001

*Note:* Mean error (CA-DA): A negative value indicated overestimation, and a positive value indicates underestimation.

Abbreviations: CA, chronological age; DA, dental age; SD, standard deviation.

**Table 2:** Comparison of the chronological and estimated dental age for Black females and Black males using the Demirjian et al. [20] method for different age ranges (years).

Age range	Black female				Black Male			
	Mean CA ± SD (years)	Mean DA for maxilla ± SD (years)	Mean DA for mandible ± SD (years)	Mean DA for maxilla and mandible ± SD (years)	Mean CA ± SD (years)	Mean DA for maxilla ± SD (years)	Mean DA for mandible ± SD (years)	Mean DA for maxilla and mandible ± SD (years)
5.00–5.99	5.58 ± 0.31	6.97 ± 0.24	7.16 ± 0.15	7.09 ± 0.20	5.54 ± 0.25	6.87 ± 0.24	7.16 ± 0.27	7.01 ± 0.26
6.00–6.99	6.44 ± 0.39	7.38 ± 0.15	7.52 ± 0.21	7.44 ± 0.18	6.32 ± 0.32	7.39 ± 0.35	7.55 ± 0.33	7.48 ± 0.32
7.00–7.99	7.46 ± 0.21	7.93 ± 0.66	8.67 ± 1.12	8.19 ± 0.83	7.44 ± 0.21	8.04 ± 0.48	8.13 ± 0.50	8.12 ± 0.44
8.00–8.99	8.44 ± 0.24	8.84 ± 0.67	9.97 ± 0.74	9.28 ± 0.58	8.40 ± 0.27	8.92 ± 0.61	9.43 ± 0.61	9.12 ± 0.56
9.00–9.99	9.54 ± 0.23	10.56 ± 0.90	11.20 ± 0.51	10.83 ± 0.65	9.49 ± 0.21	10.57 ± 1.25	10.74 ± 1.33	10.59 ± 1.26
10.00–10.99	10.54 ± 0.24	11.56 ± 0.44	11.55 ± 0.41	11.54 ± 0.39	10.56 ± 0.33	11.33 ± 0.80	12.08 ± 0.53	11.63 ± 0.60
11.00–11.99	11.38 ± 0.30	12.35 ± 0.48	12.73 ± 0.65	12.48 ± 0.47	11.49 ± 0.28	12.72 ± 0.65	13.23 ± 0.63	12.95 ± 0.39
12.00–12.99	12.40 ± 0.25	13.36 ± 0.80	13.76 + 0.72	13.55 ± 0.62	12.40 ± 0.30	14.22 ± 0.91	14.61 ± 0.81	14.38 ± 0.60
13.00–13.99	13.53 ± 0.29	14.50 ± 0.79	13.90 ± 0.97	14.20 ± 0.69	13.49 ± 0.22	15.17 ± 0.87	15.63 ± 0.51	15.54 ± 0.61
14.00–14.99	14.49 ± 0.33	15.19 ± 0.29	15.46 ± 0.58	15.32 ± 0.55	14.54 ± 0.26	15.79 ± 0.42	15.99 ± 0.03	15.98 ± 0.06
15.00–15.99	15.49 ± 0.23	15.90 ± 0.32	15.80 ± 0.42	15.86 ± 0.32	15.60 ± 0.29	16.00 ± 0.00	16.00 ± 0.00	16.00 ± 0.00
16.00–16.99	16.54 ± 0.30	16.00 ± 0.00	16.00 ± 0.00	16.00 ± 0.00	16.64 ± 0.23	16.00 ± 0.00	16.00 ± 0.00	16.00 ± 0.00

Abbreviations: CA, chronological age; DA, dental age; SD, standard derivation.

**Table 3:** Comparison of the chronological and estimated dental age for Indian females and Indian males using the Demirjian et al. [20] method for different age ranges (years).

Age range	Indian female				Indian Male			
	Mean CA ± SD (years)	Mean DA for maxilla ± SD (years)	Mean DA for mandible ± SD (years)	Mean DA for maxilla and mandible ± SD (years)	Mean CA ± SD (years)	Mean DA for maxilla ± SD (years)	Mean DA for mandible ± SD (years)	Mean DA for maxilla and mandible ± SD (years)
5.00–5.99	5.52 ± 0.27	6.49 ± 0.55	6.99 ± 0.42	6.78 ± 0.42	5.43 ± 0.30	6.62 ± 0.63	6.88 ± 0.43	6.76 ± 0.44
6.00–6.99	6.61 ± 0.30	7.25 ± 0.29	7.50 ± 0.15	7.39 ± 0.17	6.52 ± 0.18	7.15 ± 0.50	7.51 ± 0.43	7.43 ± 0.46
7.00–7.99	7.53 ± 0.34	7.72 ± 0.24	8.16 ± 0.63	7.88 ± 0.38	7.62 ± 0.28	7.84 ± 0.58	8.33 ± 0.50	8.06 ± 0.45
8.00–8.99	8.55 ± 0.26	8.53 ± 0.82	9.27 ± 0.93	8.82 ± 0.84	8.33 ± 0.22	8.32 ± 0.53	9.69 ± 0.97	8.74 ± 0.56
9.00–9.99	9.49 ± 0.23	9.72 ± 0.98	10.63 ± 1.11	10.07 ± 0.95	9.45 ± 0.31	9.49 ± 0.78	11.11 ± 0.83	10.11 ± 0.72
10.00–10.99	10.60 ± 0.30	11.45 ± 0.70	11.31 ± 0.74	11.35 ± 0.69	10.53 ± 0.34	11.30 ± 1.44	12.04 ± 0.28	11.63 ± 0.32
11.00–11.99	11.53 ± 0.31	12.35 ± 0.74	12.05 ± 0.54	12.19 ± 0.61	11.46 ± 0.27	12.46 ± 0.90	12.88 ± 0.58	12.63 ± 0.48
12.00–12.99	12.33 ± 0.36	12.98 ± 0.79	13.06 + 1.01	12.98 ± 0.77	12.42 ± 0.32	13.15 ± 0.89	14.20 ± 0.45	13.62 ± 0.64
13.00–13.99	13.59 ± 0.27	14.06 ± 0.54	14.77 ± 0.74	14.37 ± 0.41	13.67 ± 0.30	14.91 ± 0.66	15.66 ± 0.72	15.68 ± 0.66
14.00–14.99	14.59 ± 0.24	14.82 ± 0.75	15.03 ± 0.65	14.93 ± 0.65	14.50 ± 0.22	15.62 ± 0.52	15.68 ± 0.70	15.71 ± 0.62
15.00–15.99	15.45 ± 0.15	15.66 ± 0.56	15.65 ± 0.58	15.66 ± 0.49	15.41 ± 0.31	16.00 ± 0.00	16.00 ± 0.00	16.00 ± 0.00
16.00–16.99	16.66 ± 0.25	16.00 ± 0.00	16.00 ± 0.00	16.00 ± 0.00	16.51 ± 0.20	16.00 ± 0.00	16.00 ± 0.00	16.00 ± 0.00

Abbreviations: CA, chronological age; DA, dental age; SD, standard deviation.

## Data Availability

Data may be available upon written request to the authors.
